# The association between periodontal disease and the risk of myocardial infarction: a pooled analysis of observational studies

**DOI:** 10.1186/s12872-017-0480-y

**Published:** 2017-02-01

**Authors:** Shuai Xu, Mingbao Song, Yu Xiong, Xi Liu, Yongming He, Zhexue Qin

**Affiliations:** 1 Department of Stomatology, Xinqiao Hospital, Third Military Medical University, 400037 Chongqing, China; 2 Department of Cardiology, Xinqiao Hospital, Third Military Medical University, 183 Xinqiaozhengjie St., Shapingba District, 400037 Chongqing, China; 3 Department of Stomatology, Southwest Hospital, Third Military Medical University, 400038 Chongqing, China

**Keywords:** Periodontal disease, Myocardial infarction, Observational studies, Meta-analysis

## Abstract

**Background:**

Several meta-analyses have indicated that periodontal disease (PD) are related to cardiovascular diseases (CVDs). However, the association between PD and myocardial infarction (MI) remains controversial. Here we aimed to assess the association between PD and MI by meta-analysis of observational studies.

**Methods:**

PubMed, EMBASE and the Cochrane Library were searched through July, 2016. Observational studies including cohort, cross-sectional and case–control studies reporting odds ratio (OR) or relative risk (RR) with 95% confidence intervals (CIs) were included in the analysis. Either fixed or random-effects model were applied to evaluate the pooled risk estimates. Sensitivity and subgroup analyses were also carried out to identify the sources of heterogeneity. Publication bias was assessed by the Begg’s, Egger’s test and funnel plot.

**Results:**

We included 22 observational studies with 4 cohort, 6 cross-sectional and 12 case–control studies, including 129,630 participants. Patients with PD have increased risk of MI (OR 2.02; 95% CI 1.59-2.57). Substantial heterogeneity in risk estimates was revealed. Subgroup analyses showed that the higher risk of MI in PD patients exists in both cross-sectional studies (OR 1.71; 95% CI 1.07-2.73) and case–control studies (OR 2.93; 95% CI 1.95-4.39), and marginally in cohort studies (OR 1.18; 95% CI 0.98-1.42). Further, subgroup meta-analyses by location, PD exposure, participant number, and study quality showed that PD was significantly associated with elevated risk of MI.

**Conclusion:**

Our meta-analysis suggested that PD is associated with increased risk of future MI. However, the causative relation between PD and MI remains not established based on the pooled estimates from observational studies and more studies are warranted.

**Electronic supplementary material:**

The online version of this article (doi:10.1186/s12872-017-0480-y) contains supplementary material, which is available to authorized users.

## Background

Periodontal disease (PD) is defined as chronic inflammation of the supporting structure of the teeth. PD is estimated to be detectable in 20-50% of the general population and a major cause of tooth loss in adults [1]. The symptoms include bleeding gums (gingivitis), the formation of gingival pockets and bone loss (periodontitis) and finally tooth mobility caused by biofilm and calculus [2]. PD is related to many risk factors, such as inflammation, gender, smoking, genetics and life styles, which were also implicated in cardiovascular diseases (CVD) [3].

A great body of evidence has investigated the association between PD and CVD. Even though a number of meta-analyses indicated the association between PD and CVD, the cause-and-effect relationship between them was still not established with current available evidence according to an American Heart Association scientific statement [4]. Meanwhile, no specified meta-analysis by now has been reported to investigate whether PD was associated with myocardial infarction (MI).

In 1989, Mattila et al. first reported oral health including PD was related to acute MI [5]. Since then, a variety of literatures explored the nature of the association between PD and MI. Several case–control, cross-section or cohort studies have indicated a significant association between PD and MI [6–17]. However, whether the association is causal is still a matter of debate. Some other studies found no such relationship [18–24]. Given the high prevalence of PD and MI, it is of great value to determine their association.

The purpose of this study was to examine the association between PD and MI in observational studies. By pooling data from individual studies and using meta-analysis, the statistical estimates of the association between PD and incidence of MI were obtained.

## Methods

The meta-analysis was performed following the guidelines from Meta-Analysis of Observational Studies in Epidemiology (MOOSE) group [25] and the Preferred Reporting Items for Systematic Reviews and Meta-Analyses (PRISMA) statement [26].

### Eligibility criteria

Studies included for the meta-analysis if they referred to observational studies including cross-sectional, case–control, cohort and nested case–control studies. The article should estimate the association between PD and MI and provide the odds ratio (OR) or relative risk (RR) with the corresponding 95% confidence intervals (CIs).

### Search strategy

Pubmed, EMBASE and the Cochrane Library Databases were searched to identify relevant studies through July 2016. The medical subject headings (MeSH terms) as well as free text terms were used in the search strategy. The terms applied were as follows: ("periodontal disease" OR "periodontitis" OR "periodontal" OR "periodontal attachment loss" OR "periodontal pocket" OR "alveolar bone loss") AND ("myocardial infarction" OR "acute myocardial infarction" OR "acute coronary syndrome" OR "cardiovascular disease" OR "coronary heart disease" OR "unstable angina") (Additional file 1: Table S1). The search was restricted to studies conducted on human subjects and published in English and Chinese. The bibliographies of the articles found were hand searched for possible additional studies.

Two reviewers (S. Xu and X. Liu) screened the titles and abstracts of the search results and articles were excluded if they did not meet the above-mentioned inclusion criteria.

### Quality assessment

Newcastle-Ottawa Quality Assessment Scale was used to assess the quality of the observational studies, including case–control and cohort studies [27] and the Agency for Healthcare Research and Quality (AHRQ) for cross-sectional studies [28]. For case–control and cohort studies, the quality was ranked as followed: low quality = 0-4; moderate quality = 5-7; high quality = 8-9.

To rank the cross-sectional studies, the AHRQ was quantified in the following way. An item would be scored ‘0’, if it was answered ‘NO’ or ‘UNCLEAR’. If it was ‘YES’, then this item was scored ‘1’. Article quality was ranked as follows: low quality = 0-3; moderate quality = 4-7; high quality = 8-11 [29].

### Data extraction

Two reviewers (S. Xu and X. Liu) extracted the data from all included studies independently. Any discrepancies or uncertainties between the reviewers were resolved by consensus after rechecking the source and discussion with the third reviewer (Z. Qin). A data collection form was used to compile extracted study information and included the following items: first author’s surname, publication year, country of origin, sample size, population, study design, enrolled period, age ranges, PD exposure parameter, adjusted OR/RR and their 95% CI, *P* values and adjusting factors.

PD was defined to include self-report diagnosis, or any measure of disease according to clinical, radiographic and microbiological assessment (including pocket probing depth, attachment loss, bleeding on probing, plaque index, gingival index, X-ray and microbiological results). At the very beginning, all related OR/RR based on different PD assessments were extracted. The one selected for final meta-analysis was according to the frequency of each OR/RR in all included studies. The order in this study was clinical attachment loss (CAL), pockets deep and periodontal bone loss.

### Data analysis

The OR was used in these studies, while RR and hazard ratio (HR) were considered equivalent. For one study that reported stratified OR/RR in different population, we considered each subgroup analysis as an independent study. Before pooling the data, ORs were transformed into their natural logarithm to stabilize their variance, normalize the distribution and then statistically pooled [30].

The heterogeneity among studies was evaluated by Cochrane Q test and quantified as I^2^ metric. For the Q statistic, a *P*-value <0.1 was considered statistically significant heterogeneity. For I^2^ statistic, a value of zero was considered no heterogeneity, 25%-49% low, 50%-74% moderate, and above 75% high, respectively. To explore possible reasons for heterogeneity and test the robustness of the association, subgroup analyses were performed based on study design, methodological quality, gender, locations and PD exposure assessment parameters. When obvious heterogeneity across studies exists, the random-effect model was used to calculate the pooled estimates. Otherwise, the fixed-effect model was applied.

Publication bias was assessed by both Begg’s rank correlation test and Egger’s linear regression test. Funnel plots were also generated to assess the publication bias.

STATA statistical software, version 11.0 (STATA Corporation, College Station, TX, USA) was used for analysis. *P* values were 2-sided and *P* < 0.05 was considered statistically significantly otherwise indicated.

## Results

### Literature search

**Fig. 1:**
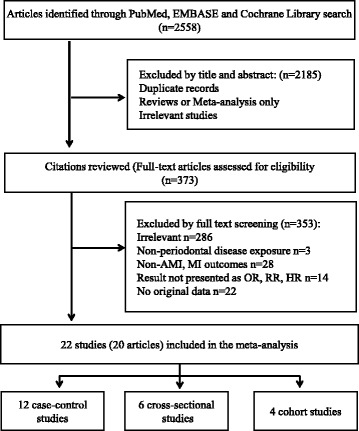
Flow Chart for study selection

Initially, a total of 2558 articles were identified from PubMed (2041 articles), EMBASE (415 articles) and the Cochrane Library Database (102 articles) search. Of these citations, after evaluating the titles and abstracts, 373 studies were chosen for further review by reading the full text. Among them, 353 literatures were excluded because 286 articles were irrelevant, 14 had no OR/RR values, 28 had no clearly MI endpoint, 3 were not treated PD as exposure and 22 had no original data. Finally 22 studies from 20 articles representing 129,630 participants were included in our meta-analysis [6–24, 31]. See Fig. 1 for the flow chart of the study selection process.

### Study characteristics

The characteristics of each study were demonstrated in Table 1, with adjusted covariates of each study presented. Four prospective cohort studies, 6 cross-sectional and 12 case–control studies evaluated the association between PD and MI. Case–control studies included a total of 2912 cases of MI and 3291 controls [6–15, 24]. All studies were published from 1996 to 2016. The reported age ranged from 23 to 83 years. The enrolled years of patients varied from 1982 to 2014. Among 22 studies, 8 were carried out primarily in America [8, 9, 16, 18, 21–23], 5 studies were conducted in Asian countries [12, 13, 20, 31] and 9 studies were from European countries [6, 7, 10, 11, 14, 15, 17, 19, 24]. Sixteen articles reported the OR values between PD and MI, 2 articles gave the HR and 2 articles presented with RR values. In 2 of the studies, OR/RR was less than one [20, 24]. Only two studies did not perform the adjustments for potential confounders [10, 19]. Fourteen studies showed PD was associated with MI [6–17, 31], while 8 studies demonstrated PD was not related to MI [18–24, 31]. PD exposure parameters varied among studies including clinical attachment loss (CAL), probing depth, deep pockets, and periodontal bone loss. The quality of the studies was high overall, without remarkable limitations identified (Additional file 2: Table S2; Additional file 3: Table S3).

**Table 1:** The characteristics of the studies included in the analysis

Cohort studies										
Author, year	Country	Study design	Sample size (M/F)	Population	Years enrolled (Follow-up)	Age (years)	Exposure	OR/RR (95% CI)	*P* value	Adjusting factors
Joshipura KJ et al., 1996 [21]	USA	Prospective cohort study	44119 male	757 cases	1986-1992 (6)	40-75	PD	1.04 0.86-1.25	NS	Age, BMI, exercise, smoking, alcohol, family history of MI, vitamin E
Howell TH et al., 2001 [22]	USA	Prospective cohort study	2653 male	797 cases	1982-1995 (12.3)	40-84	PD	1.01 0.82-1.24	NS	Age, aspirin, β-carotene treatment, smoking, alcohol, HT, BMI, DM, physical activity, family history of MI
Dorn JM et al., 2010 [23]	USA	Prospective cohort study	884 (668/216)	154 cases	1996-2004 (2.9)	54.5 ± 8.4	CAL	1.48 0.95-2.31	NS	Age, gender, education, DM, EF, HT, physical activity, cholesterol, lipid-lowering medication, BMI, fruit/vegetable intake, CK-MB
Yu YH et al., 2015 [16]	USA	Prospective cohort study	39863 female	642 MI	1992-1995 (15.7)	48.7-60.3	PD	1.39 1.17-1.64	<0.001	Age
Cross-sectional studies										
Author, year		Country	Sample size (M/F)	Population	Years enrolled	Age (years)	Exposure	OR/RR (95% CI)	*P* value	Adjusting factors
Bazile A et al.,2002 [18]		USA	80 (48/32)	50 CHD (20 MI)/30 control	2002	23-83 (median 54)	CAL	1.23 0.29-5.23	0.775	Age, gender
Buhlin K et al., 2002 [19]		Sweden	1577	1577 (27 MI)	1998	41-84	Deep pockets	1.32 0.51–3.38	0.57	NS
Holmlund A et al., 2006 [17]		Sweden	4254 (1866/ 2388)	3352 case/902 control	1976-2000	53 ± 14	Periodontal Bone Loss	2.69 1.12-6.46	0.03	Age, gender, smoking
Senba T et al., 2008 [31]		Japan	6,816 M	(MI = 25)	2004	NS	PD	2.34 1.05-5.23	NS	Age
Senba T et al., 2008 [31]		Japan	23,088 F	(MI = 16)	2004	NS	PD	1.76 0.64-4.88	NS	Age
Sujal M. Parkar et al., 2013 [20]		India	60 (42/18)	30 AMI/30 Control	2013	54.3 ± 11.0 (case); 53.1 ± 10.5 (control)	Community periodontal index	0.224 0.03-1.68	0.15	Age, sex, smoking, alcohol, BMI
Case–control studies										
Author, year		Country	Sample size (M/F)	Population	Years enrolled	Age (years)	Exposure	OR/RR (95% CI)	P value	Adjusting factors
Persson GR et al., 2003 [6]		Sweden	160 (138/22)	80AMI/80control	2003	63.4 ± 8.9 (case) 61.9 ± 9.1 (control)	CAL	14.1 5.8-34.4	<0.0001	Smoking
Cueto A et al., 2005 [7]		Spain	149 (89/60)	72AMI/77control	2002	62.5 ± 9.9 (case) 58.5 ± 10.2 (control)	CAL	3.31 1.42–7.71	0.005	Sex, age, smoking, HT, DM, exercise, cholesterol
Andriankaja OM et al., 2006 [8]		USA	1337 (765/572)	537MI/800control	1997-2001	54.6 ± 8.5 (case) 55.0 ± 0.0 (control)	CAL	2.77 1.95-3.94	<0.001	Age, gender, HT, DM, cholesterol, smoking
Andriankaja OM et al., 2007 [9]		USA	782 M	415AMI/367control	1997-2001	54.5 ± 8.5 (case) 56.5 ± 10.2 (control)	CAL	1.34 1.15–1.57	<0.001	Age, BMI, HT, physical activity, cholesterol, DM, smoking
Andriankaja OM et al., 2007 [9]		USA	593 F	120AMI/473Control	1997-2001	55.5 ± 8.7 (case) 54.1 ± 9.8 (control)	CAL	2.08 1.47–2.94	<0.001	Age, BMI, physical activity, HT, cholesterol, DM, smoking
Renvert S et al., 2010 [10]		Sweden	324 (287/370)	165ACS/159control	2002-2007	31-87	PD	10.3 6.1-17.4	NS	NS
Holmlund A et al., 2011 [11]		Sweden	200 (160/40)	100AMI/100control	2011	57.1 ± 5.5 (case) 57.9 ± 5.2 (control)	Pockets depth	4.61 1.52-13.94	0.0069	Age, gender, CRP, HT, smoking, IL6, cholesterol, HDL-c, DM, TG, BMI, education,
Khosravi Samani M et al., 2013 [12]		Iran	123	60AMI/63control	2013	54.97 ± 9.68 (case) 55.89 ± 11.9 (control)	CAL	8.79 2.36-32.66	0.001	Age, DM
Li P et al., 2013 [13]		China	155 (113/42)	103AMI/52control	2013	68 (41-84) (case) 62 (42-78)(control)	CAL	4.89 1.26-18.94	0.02	HT, total cholesterol HDL, BMI, LDL
Willershausen I et al., 2014 [24]		Germany	497 (380/117)	248AMI/249control	2007-2011	62.3 (51-83)	PD	0.879 0.527-1.466	0.622	Gender, age, smoking
Kodovazenitis G, et al., 2014 [14]		Greece	306 (218/88)	204MI/102 control	2007-2009	64.7 ± 12.9 (case) 64.2 ± 10.1 (control)	CAL	2.27 1.22-4.35	0.01	Age, gender, HT, total cholesterol, smoking
Rydén L et al., 2016 [15]		Sweden	1610 (1308/302)	805MI/805control	2010-2014	62.5 ± 8	PD	1.28 1.03-1.60	NS	DM, smoking, education, marital status

*BMI* body mass index, *CAL* clinical attachment loss, *CI* confidence interval, *CK-MB*, creatine phosphokinase-myocardial band, *CRP* C-response protein, *DM* diabetes mellitus, *EF* ejection fraction, *HDL-c* high-density lipoprotein cholesterol, *HT* hypertension, *MI* myocardial infarction, *NS* not specified, *OR* odd ratio, *PD* periodontal disease, *RR* relative risk, *TG* triglycerides

If the studies specified the PD exposure parameters, the detailed PD parameters would be displayed in this table. Otherwise, the PD would be considered as exposure factor

### Meta-analysis

**Fig. 2:**
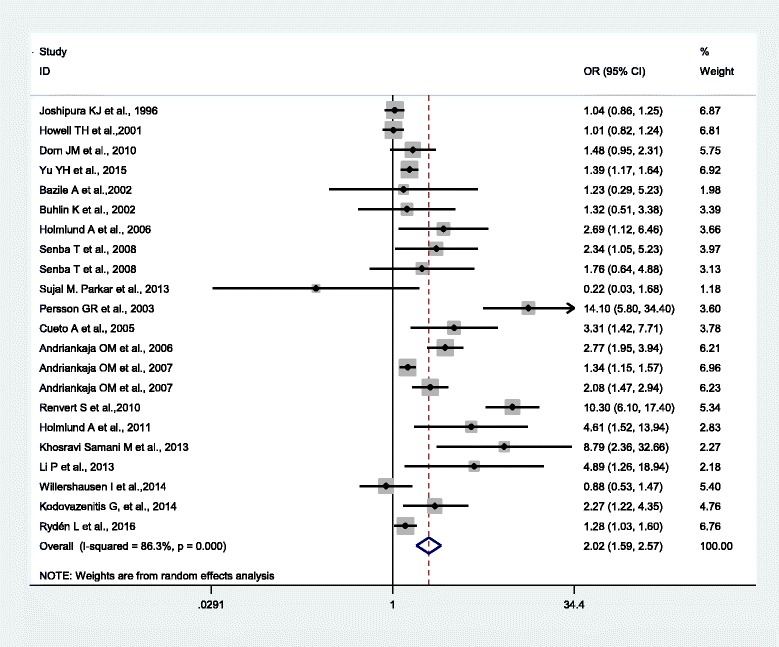
Meta-analysis of observational studies on periodontal disease and myocardial infarction in a random-effect model. OR, odds ratio; RR, relative ratio; CI, confidence interval

**Fig. 3:**
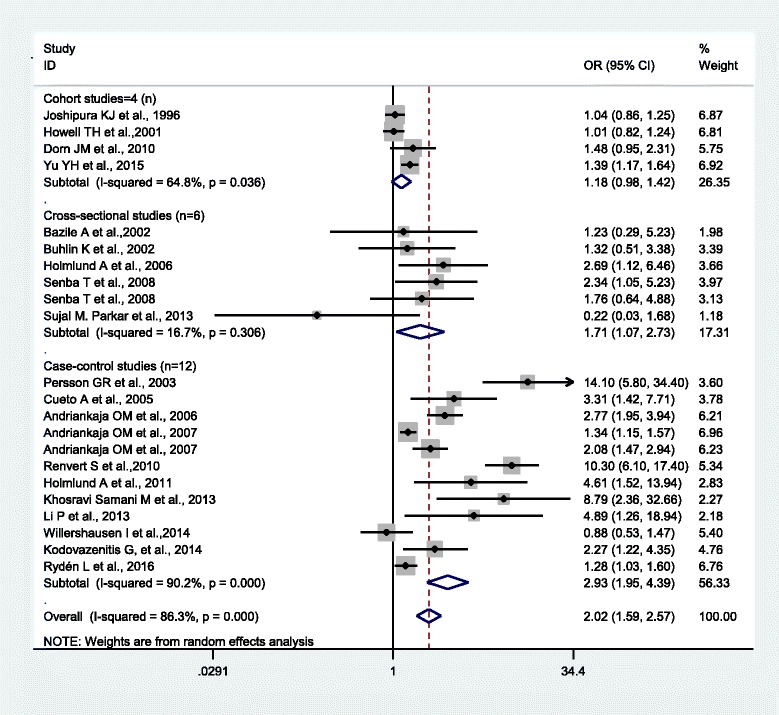
Association between periodontal disease and myocardial infarction in a random-effect model meta-analyses by study design. OR, odds ratio; RR, relative ratio; CI, confidence interval

The OR/RR from 22 studies were pooled and analyzed as presented in Fig. 2. Overall, the PD had statistically significant correlation with MI risk (OR = 2.02, 95% CI 1.59-2.57). A severe statistical heterogeneity among studies was revealed (Q = 152.77, *P* < 0.001, I^2^ = 86.3%). Sensitivity analysis showed two studies contributed most to the heterogeneity [6, 10]. Although the sensitivity remains marked, the Q value and I^2^ decreased to 70.68, and 73.1%, respectively. In the subgroup analysis by type of study (Fig. 3), cohort studies showed marginally increased risk of MI (RR 1.18, 95% CI 0.98-1.42; I^2^ = 64.8%, n = 4) [16, 21–23], while PD increased the risk of MI in both cross-sectional studies (OR 1.71, 95% CI 1.07-2.73; I^2^ = 16.7%, n = 6) [17–20, 31] and case–control studies (OR 2.93, 95% CI 1.95-4.39; I^2^ = 90.2%, n = 12) [6–15, 24].

**Fig. 4:**
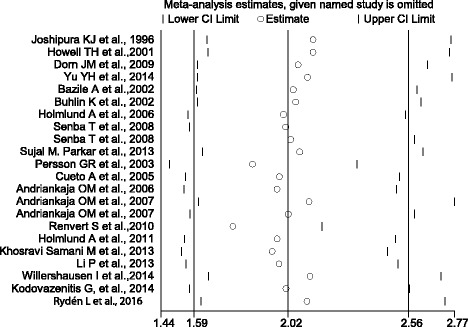
Sensitivity analysis on meta-analysis between periodontal disease and myocardial infarction

By removing each study at a time, sensitivity analysis was conducted to determine the influence of each study on the pooled OR. By omitting one study in turn, the pooled OR and 95% CI was not modified significantly (Fig. 4). This sensitivity analysis indicated that the results of this meta-analysis were stable and reliable.

**Fig. 5:**
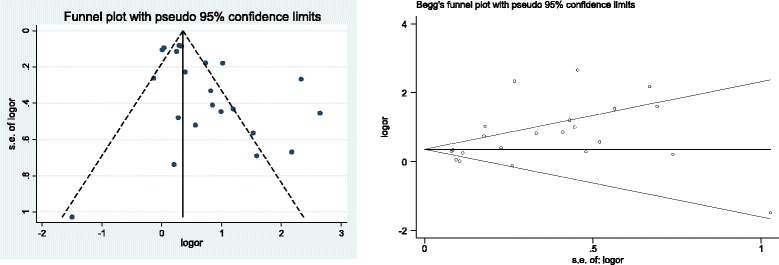
Funnel plot of the included studies for publication bias

Additionally, publication bias was observed from Egger’s Test (*P* = 0.013), Begg’s Test (*P* = 0.195), and Funnel plot (Fig. 5). Trim and fill algorithm analysis was performed and the analysis showed “no trimming performed; data unchanged”.

### Subgroup analysis

To examine the stability of the studies, subgroup analyses were carried out based on several factors. Patients with PD had increased risk of MI across most subgroups by location, PD exposure parameters and study quality (Table 2). In the gender-specific subgroup, 3 female pooled studies showed an increased risk of MI (OR = 1.64, 95% CI 1.20-2.25) [8, 18, 23], while the pooled results in the 4 male studies were marginally increased (OR = 1.18, 95% CI 0.96-1.44) [9, 21, 22, 31]. Overall, subgroup analyses by various factors demonstrated that patients with PD have a higher risk to develop MI.

**Table 2:** Subgroup analysis

Group	Number of studies	Pooled RR	95% CI	*P* (heterogeneity)	I^2^ (%)
All studies	22	2.02	1.59-2.57	0.000	86.3%
Location					
America	8	1.44	1.16-1.78	0.000	81.1%
Asia	9	2.93	1.52-5.65	0.000	90.7%
Europe	5	2.44	1.01-5.86	0.0033	61.8%
Gender					
Female	3	1.64	1.20-2.25	0.117	53.4%
Male	4	1.18	0.96-1.44	0.026	67.6%
Exposure					
CAL	10	2.66	1.80-3.94	0.000	82.5%
Others	12	1.65	1.20-2.27	0.000	87.3%
Study quality					
High	12	2.29	1.61-3.25	0.000	91.0%
Low and moderate	10	1.66	1.20-2.29	0.001	66.5%
Number of participants					
> 1000	9	1.46	1.16-1.83	0.000	76.8%
< 1000	13	2.69	1.68-4.30	0.000	88.3%

### Discussion

In this study, we identified a statistical positive correlation between PD and MI. Patients with PD have approximately a 1-fold increase in risk for MI. Given the high prevalence of PD in population [32], this increase might mean a profound public health impact.

Several meta-analyses have reported that subjects with PD have higher odds and higher risks of developing cardiovascular diseases [33–38]. Teeuw et al pooled 25 studies on treatment of periodontitis and demonstrated intervention of PD improved the atherosclerotic profile [39], but the direct evidence of reduction in the risk of cardiovascular events associated with periodontitis treatment remains lacking. Further, a meta-analysis reported that patients with periodontitis compared to controls have increased arterial stiffness [39]. However, the only two interventional studies contradicted with each other on the effects of periodontal treatment on arterial stiffness [40]. Therefore, PD appears to be associated with increased risk of CVD, but the causal relation is still not well established.

Myocardial infarction is one of the most life-threating cardiovascular diseases. Some investigations mentioned the relationship between PD and MI, while dispute exists. In 1996, Joshipural KJ’s group followed up a cohort including 44119 males for 6 years and found no overall association between PD and fatal or non-fatal MI in this population [21]. This negative result was further echoed by some other prospective cohort studies [22, 23]. However, a large body of evidence showed there was an association between PD and MI. Very recently a large case–control study indicated that the risk of MI was significantly increased in patients with PD even after adjustment for confounding factors [15]. These disputes required further systemic analyses on the relation between MI and PD. However, to the best of our knowledge, no meta-analyses specified the relation between PD and MI.

In this study, we have showed an overall increased risk for MI in PD patients. The subgroup meta-analyses by study design showed that PD was associated with MI in both cross-sectional and case–control studies, while the relation in the cohort studies was marginal. Likewise, the subgroup analyses by country, PD exposure, participant number and study quality consistently showed an apparent increased risk of MI in PD subjects. Moreover, a marginal but non-significant increase of risk was present in male population. These non-significant findings exist in cohort studies and male participants might be attributed to the small number of studies included in the subgroup meta-analyses. Intriguingly, Rydén L group in their case–control study specified that the risk of a first MI was significantly increased in patients with PD. [15] However, most of the studies did not clarify the MI occurrence which presumably might be first ever MI. Renvert S et al also reported the relation between PD and recurrent MI [10]. First and recurrent MI differentiation would better illustrate the predication value of PD. Regarding limited data, the PD predication efficacy comparison between first and recurrent MI remains unknown.

Although not fully illustrated, several mechanisms were speculated to be involved in such a relation between PD and MI. The periodontal pathogen burden and its by-products might not only contribute to the development of atherosclerosis but also the rupture of atherosclerotic plaques and occurrence of MI. Dozens of literature has demonstrated that periodontal bacteria are detected in from atherosclerotic plaques. Ohki T et al. further suggested that three species of periodontal bacteria were present in the thrombi of patients with acute MI [41]. This finding suggested that periodontal bacteria might play a role in plaque inflammation and instability. Meanwhile, periodontal bacteria and the lipopolysaccharide from them could trigger inflammatory factor release, such as tumor necrosis factor α, interleukins, whose roles are well established in atherogenesis and rupture of developed lesions [42]. Further, C-reactive protein (CRP), a non-specific acute-phase factor participating in the systemic response to inflammation, helps to improve the risk prediction of cardiovascular events. Patients with PD also have increased serum levels of CRP relative to unaffected subjects [43]. The level of infection with periodontal pathogens is positively correlated with CRP level [44]. These evidence suggests that CRP might underlie the relation between PD and MI. In addition, platelet aggregation and thromboembolic events could be activated by periodontal pathogens themselves and many other cytokines [38].

Heterogeneity among studies was revealed in this analysis, although the relation between PD and MI has similar tendency in the overall and subgroup analysis. The significant heterogeneity was also reported in meta-analysis on PD and cardiovascular diseases [45, 46]. By nature, the diagnosis of PD was quite variable and included a series of parameters, such as attachment loss, bone loss pocket, probing depth, bleeding on probing, plaque index, gingival index, X-ray and microbiological results, teeth loss and the Russell periodontal index. Different PD assessment resulted in discrepancy when analyzing the relation between PD and MI. Renvert S’ group compared 5 PD parameters and their combination, founding that comprehensive PD data should be used in studies of associations between periodontitis and heart diseases [47]. Many other risk factors including age, smoking, and diabetes are common in both PD and MI. These factors could be taken into consideration as confounding factor. Different confounding factors were adjusted as demonstrated in Table1. This different adjustment could also affect the adjusted OR/RR when investigating the relation between PD and MI. Moreover, study design, study quality, number of participants and location were examined to find the sources of heterogeneity. The subgroup analysis showed these factors did not appreciably change the heterogeneity among studies. Further, sensitivity analyses showed no alterations to the pooled OR by omitting each study. Together, the significant heterogeneity should be considered when interpreting the results in this study, although the strong relation between PD and MI are observed.

Most of the studies that exclude for the meta-analysis in this study also supported a positive association between PD and MI. A population-based study showed periodontitis was associated with an increased risk of major adverse cardiac events including MI occurrence [48]. Other studies also indicated that PD may be associated with MI in Turkish and Iran population [49, 50]. Vice versa, there was higher prevalence of edentulousness and advanced PD in the hospitalized MI group than that in the non-MI group [51]. A case–control study also showed MI patients exhibited an unfavorable dental chronic infection compared with healthy patients. These data from the other angle suggests an association between PD and MI.

Several limitation of this meta-analysis should be noted. First, limited by the nature of observational studies, the causality between PD and MI cannot be inferred from our findings. Second, as aforementioned, definitions for PD in each study included in the analysis were not identical. Although we set rules when extracting data, this discrepancy in the assessments for PD seemed not to be eliminated nor even decreased obviously. Additionally, the differences in the study design, population and criteria to measure the exposure may weaken the evidence and lead to considerable heterogeneity. Although further analysis were performed, the sources of the heterogeneity remained not clear. The meta-regression analysis might further explain the source of heterogeneity. For the paucity of the data, these analyses were not conducted. Finally, the concrete mechanisms to explain the PD and MI relation still need further investigation, though possible mechanisms were discussed above. Further prospective studies are warranted to establish the association between PD and MI, while it might also be helpful to confirm the relation with randomized, controlled interventional trials including medication to control the pain and inflammation and surgery to remove the inflamed tissue and reduce the bone damage.

## Conclusion

In conclusion, our meta-analysis yielded a statistically significant association between PD and MI. Subgroup analyses also confirmed the elevated risk for MI in PD subjects, although heterogeneity should be noted. More studies including large-scale prospective cohort studies and randomized controlled trials are warranted.

## Additional files

Additional file 1: Table S1. Search Strategy. (PDF 66 kb)
Additional file 2: Table S2. Quality scores of case–control and cohort studies using Newcastle-Ottawa Scale. (PDF 37 kb)
Additional file 3: Table S3. Quality scores of cross-sectional studies using Agency for Healthcare Research and Quality Scale. (PDF 34 kb)

## Abbreviations

AHRQ: Agency for healthcare research and quality
CAL: Clinical attachment loss
CIs: Confidence intervals
CRP: C-reactive protein
CVDs: Cardiovascular diseases
HR: Hazard ratio
MI: Myocardial infarction
MOOSE: Meta-analysis of observational studies in epidemiology
OR: Odds ratio
PD: Periodontal disease
PRISMA: Preferred reporting items for systematic reviews and meta-analyses
RR: Relative risk
