# Supplementary material for: The association between periodontal disease and the risk of myocardial infarction: a pooled analysis of observational studies
Source: BMC Cardiovasc Disord. 2017 Feb 1;17:50. doi: 10.1186/s12872-017-0480-y (PMC5286862; doi:10.1186/s12872-017-0480-y)
Supplement: Additional file 1: Table S1. — Search Strategy. (PDF 66 kb) [file 12872_2017_480_MOESM1_ESM.pdf]

Table S1. Search Strategy.

Search Terms
1. “periodontal disease [MeSH Terms]” OR “periodontitis [MeSH Terms]” OR “periodontal[free term]” OR “periodontal attachment loss [MeSH Terms]” OR “periodontal pocket [MeSH Terms]” OR “alveolar bone loss [MeSH Terms]”
2. “myocardial infarction[MeSH Terms]” OR “acute myocardial infarction[MeSH Terms]” OR “acute coronary syndrome[MeSH Terms]” OR “cardiovascular disease [MeSH Terms]” OR “coronary heart disease [MeSH Terms]” OR “unstable angina [MeSH Terms]”
3. 1 AND 2
