# Supplementary material for: The association between periodontal disease and the risk of myocardial infarction: a pooled analysis of observational studies
Source: BMC Cardiovasc Disord. 2017 Feb 1;17:50. doi: 10.1186/s12872-017-0480-y (PMC5286862; doi:10.1186/s12872-017-0480-y)
Supplement: Additional file 2: Table S2. — Quality scores of case–control and cohort studies using Newcastle-Ottawa Scale. (PDF 37 kb) [file 12872_2017_480_MOESM2_ESM.pdf]

Table S2. Quality scores of case-control and cohort studies using Newcastle-Ottawa Scale.

Methodological quality of studies included in the final analysis based on the Newcastle-Ottawa Scale for assessing the quality of Case-control studies and Prospective Cohort studies									
	Selection				Comparability	Exposure			
**Case-control studies(n=12)**	Adequate definition of cases	Representativeness of cases	Selection of controls	Definition of controls	Control for important factor or additional factor	Ascertainment of exposure(blinding)	Same method of ascertainment for participants	Nonresponse rate	Total
Persson GR et al., 2003	1	1	1	1	2	1	1	1	9
Cueto A et al., 2005	0	1	0	0	2	1	1	1	6
Andriankaja OM et al., 2006	1	0	1	1	2	1	1	1	8
Andriankaja OM et al., 2007(Male)	1	1	1	1	2	1	1	1	9
Andriankaja OM et al., 2007(Female)	1	1	1	1	2	1	1	1	9
Renvert S et al.,2010	1	1	1	1	2	1	1	1	9
Holmlund A et al., 2011	1	1	1	1	2	1	1	1	9

Khosravi Samani M et al., 2013	1	1	0	0	2	1	1	1	7
Li P et al., 2013	1	1	1	1	2	1	1	1	9
Willershausen I et al.,2014	1	1	1	1	2	1	1	1	9
Kodovazenitis G, et al., 2014	1	1	0	1	2	1	1	1	8
Rydén L et al., 2016	1	1	1	1	2	1	1	1	9
	Selection				Comparability	Outcome			
**Prospective Cohort studies(n=4)**	Representativeness of the exposed cohort	Selection of non-exposed cohort	Ascertainment of exposure	Outcome of interest was not present at start of study	Control for important factor or additional factor	Assessment of outcome	Follow-up long enough for outcomes to occur	Adequacy of follow-up of cohorts	Total
Joshipura KJ et al., 1996	0	1	0	1	2	1	1	1	7
Howell TH et al.,2001	1	1	0	1	2	1	1	1	8
Dorn JM et al., 2010	0	1	1	1	2	1	1	1	8
Yu YH et al., 2015	0	1	0	1	2	1	1	1	7
