# Supplementary material for: The association between periodontal disease and the risk of myocardial infarction: a pooled analysis of observational studies
Source: BMC Cardiovasc Disord. 2017 Feb 1;17:50. doi: 10.1186/s12872-017-0480-y (PMC5286862; doi:10.1186/s12872-017-0480-y)
Supplement: Additional file 3: Table S3. — Quality scores of cross-sectional studies using Agency for Healthcare Research and Quality Scale. (PDF 34 kb) [file 12872_2017_480_MOESM3_ESM.pdf]

Table S3. Quality scores of cross-sectional studies using Agency for Healthcare Research and Quality Scale.

Methodological quality of studies included in the final analysis based on the Agency for Healthcare Research and Quality(AHRQ)for assessing the quality of cross-sectional studies(n=6)∗												
Cross-sectional studies (n=6)	1	2	3	4	5	6	7	8	9	10	11	Score
Bazile A et al.,2002	Yes	Yes	No	Unclear	Unclear	Yes	No	Yes	No	Yes	No	5/11
Buhlin K et al., 2002	Yes	No	No	Yes	Unclear	No	No	Yes	No	Yes	No	4/11
Holmlund A et al., 2006	Yes	No	Yes	Unclear	Unclear	Yes	No	Yes	No	Yes	No	5/11
Senba T et al., 2008(Male)	Yes	No	No	Yes	Unclear	No	Yes	Yes	No	Yes	No	5/11
Senba T et al., 2008(Female)	Yes	No	No	Yes	Unclear	No	Yes	Yes	No	Yes	No	5/11
Sujal M. Parkar et al., 2013	Yes	Yes	No	Unclear	Unclear	Yes	No	Yes	No	Yes	No	5/11

∗1.Define the source of information(survey, record review)2.List inclusion and exclusion criteria for exposed and unexposed subjects (case and controls) or refer to previous publications 3.Indicate time period used for identifying patients 4.Indicate whether or not subjects were consecutive if not population-based 5.Indicate if evaluators of subjective components of study were masked to other aspects of the status of the participants 6.Describe any assessments undertaken for quality assurance purposes(e.g., test/retest of primary outcome measurements) 7.Explain any patient exclusion from analysis 8.Describe how confounding was assessed and/or controlled 9.If applicable, explain how missing data were handled in the analysis 10.Summarize patient response rates and completeness of data collection 11.Clarify what follow-up, if any, was expected and the percentage of patients for which incomplete data or follow-up was obtained.
